# Osteolysis of the Greater Trochanter Caused by a Foreign Body Granuloma Associated with the Ethibond® Suture after Total Hip Arthroplasty

**DOI:** 10.1155/2017/6082302

**Published:** 2017-01-31

**Authors:** Keiji Kamo, Hiroaki Kijima, Koichiro Okuyama, Nobutoshi Seki, Shin Yamada, Naohisa Miyakoshi, Yoichi Shimada

**Affiliations:** ^1^Department of Orthopedic Surgery, Akita Rosai Hospital, Odate, Japan; ^2^Akita Hip Research Group (AHRG), Akita, Japan; ^3^Department of Orthopedic Surgery, Akita University Graduate School of Medicine, Akita, Japan

## Abstract

The present case shows a case of progression of osteolysis of the greater trochanter caused by a foreign body granuloma associated with the number 5 Ethibond suture in cementless THA with the direct lateral approach that was completely healed by removal of the Ethibond suture. A 55-year-old Japanese woman with secondary osteoarthritis caused by acetabular dysplasia underwent left cementless THA with the direct lateral approach. After setting of the total hip prosthesis, the gluteus medius muscle and vastus lateralis muscle were reattached to the greater trochanter through two bone tunnels using number 5 Ethibond EXCEL sutures. The left hip pain disappeared after surgery, but the bone tunnels enlarged gradually and developed osteolysis at 10 weeks. The removal of the Ethibond sutures and debridement improved the osteolysis. Histological examination showed the granuloma reaction to a foreign body with giant cell formation. The Ethibond suture has the lowest inflammatory tissue reaction and relatively high tension strength among nonabsorbable suture materials. However, number 5 Ethibond has the potential to cause osteolysis due to a foreign body granuloma, as in the present case.

## 1. Introduction

The direct lateral approach to the hip was described by Hardinge in 1982. With this approach, the anterior half of the gluteus medius muscle and the vastus lateralis muscles are detached from the greater trochanter [1]. In this case, the gluteus medius should be reattached to the greater trochanter to prevent a positive Trendelenburg sign after surgery. Several techniques of soft tissues reattachment in total hip arthroplasty (THA) have been reported [2–5]. The technique of soft tissues reattachment by nonabsorbable suture through bone tunnels is often used in THA with the direct lateral approach and posterior approach [2–4]. Ethibond suture has characteristics of both the lowest inflammatory tissue reaction and relatively high tension strength among nonabsorbable suture materials. Therefore, Ethibond suture is preferably used for reattachment of the abductor muscles, the capsule, and the external rotators in THA. Osteolysis of the greater trochanter associated with the Ethibond suture after THA is very rare. A case of osteolysis of the greater trochanter caused by a foreign body granuloma associated with number 5 Ethibond suture, which was used to reattach the gluteus medius to the greater trochanter through bone tunnels in THA, is presented. Written informed consent was obtained from the patient for publication of this case report.

## 2. Case Presentation

**Figure 1:**
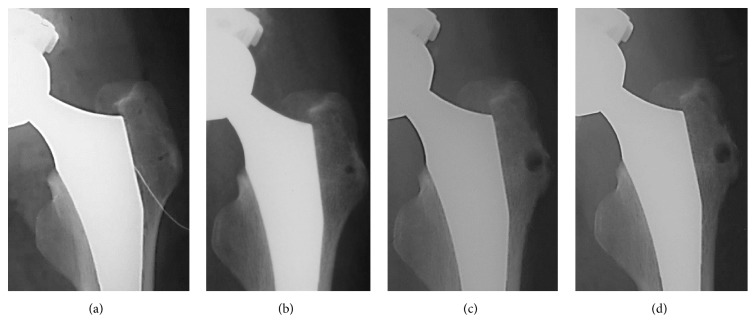
Radiographs of the left hip joint after THA. (a) The initial radiograph after THA. (b) At 4 weeks after THA. (c) At 8 weeks. (d) At 10 weeks.

**Figure 2:**
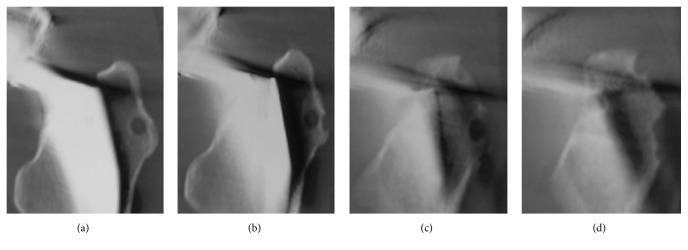
Coronal view of computed tomography showing osteolysis clearly ((a)–(d)).

**Figure 3:**
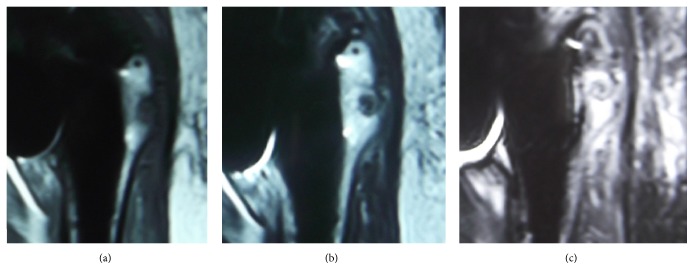
Magnetic resonance imaging at 10 weeks after THA. (a) T1-weighted image. (b) T2 star-weighted image. (c) T2 Short-TI Inversion Recovery (STIR) image.

**Figure 4:**
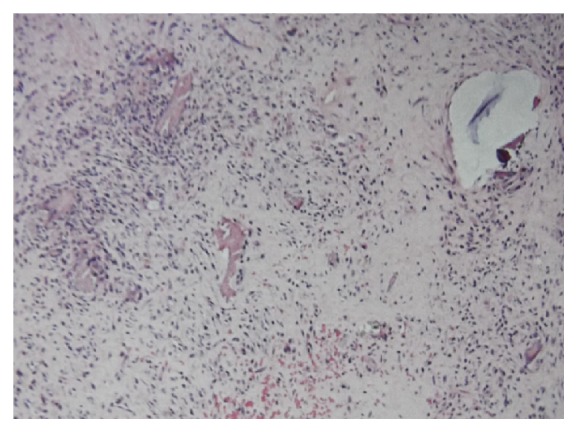
Histological examination showing the foreign body reaction granuloma with giant cell formation and proliferation of small blood vessels (hematoxylin and eosin stain; magnification: 100x).

A 55-year-old Japanese woman with secondary osteoarthritis caused by acetabular dysplasia underwent left cementless THA with the direct lateral approach. The operation was performed in the lateral position. The anterior half of the gluteus medius muscle and the vastus lateralis muscle were detached from the greater trochanter. After setting of the total hip prosthesis, the gluteus medius muscle and vastus lateralis muscle were reattached to the greater trochanter through two bone tunnels using number 5 Ethibond EXCEL (ETHICON, Johnson & Johnson, Tokyo, Japan) sutures. The left hip pain disappeared after surgery, but the bone tunnels were slightly enlarged on the radiograph 4 weeks after operation. They enlarged gradually and developed osteolysis of 10 mm in diameter at 10 weeks in the radiographs (Figure 1). Computed tomography (CT) showed the osteolysis clearly (Figure 2). Magnetic resonance imaging (MRI, 1.5 T Magnetom Symphony a Tim System, Siemens, Germany) showed the mass at the greater trochanter as low intensities on both T1-weighted images and T2 star-weighted images. The area surrounding the mass had low intensities on T1-weighted images and high intensities on T2 star-weighted images (Figure 3). The patient had no pain, and inflammatory sign was not observed on the operated site. There was no sign of infection on blood biochemical examinations. The removal of the Ethibond sutures and debridement were performed to prevent progressive osteolysis and a pathological fracture of the greater trochanter. At the time of the surgery, granulation tissue was seen surrounding the Ethibond sutures in the greater trochanter. The Ethibond sutures and the granulation tissues were completely removed. The gluteus medius muscle was repaired using absorbable number 2 Vicryl Plus® (ETHICON, Johnson & Johnson, Tokyo, Japan) sutures. Histological examination showed the granuloma reaction to a foreign body with giant cell formation and proliferation of small blood vessels (Figure 4).

**Figure 5:**
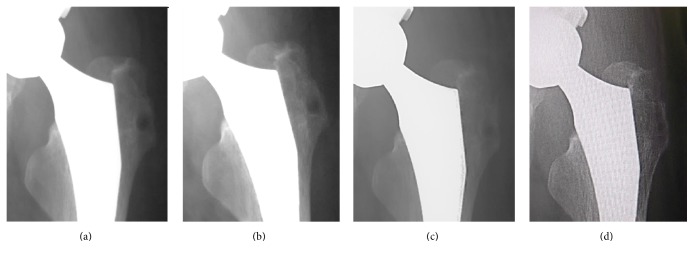
Radiographs of the left hip joint after the removal of the Ethibond suture and the granulation tissue. Bone tunnels gradually decrease and disappear. There is no evidence of osteolysis of the greater trochanter at 10 months after the second surgery. (a) At 1 month after the second surgery. (b) At 2 months. (c) At 3 months. (d) At 10 months.

After the second surgery, the bone tunnels gradually decreased and disappeared. There was no evidence of osteolysis of the greater trochanter in the radiograph at 10 months after the second operation (Figure 5). The patient can walk without pain and without a Trendelenburg sign.

## 3. Discussion

The present case is the first report of progression of osteolysis of the greater trochanter caused by a foreign body granuloma associated with the Ethibond suture in cementless THA that was completely healed by removal of the Ethibond suture. The technique of repairing muscle tendon units and ligaments by nonabsorbable suture through bone tunnels is widely used in orthopedic surgery. In THA, this technique is useful when reattaching abductor muscles to the greater trochanter in the direct lateral approach and for repairing the capsule and external rotator muscles in the posterior approach.

Ethibond is a nonabsorbable, braided surgical suture. It consists of high molecular weight, long chain, and linear polyesters with recurrent aromatic rings as an integral component and is covered with polybutylate [6]. Because of its coverage, it causes lesstissue reaction and has better mechanical properties compared with the uncovered braided polyesters [7]. Therefore, Ethibond is often used for reattachment of the abductor muscles, the capsule, and the external rotators in THA [3, 4].

In the present case, the second surgery and debridement were performed to prevent progressive osteolysis of the greater trochanter caused by the granuloma associated with the number 5 Ethibond suture. There have been few reports of osteolysis caused by the Ethibond suture. Kundra et al. reported 27 cases of cemented THA that had osteolysis of the greater trochanter following reattachment of hip abductors using number 5 Ethibond sutures through bone tunnels [4]. These patients displayed a predominantly osteolytic pattern of bone reaction around the greater trochanter bone tunnels. Histological examination of the specimen showed chronic inflammation with a giant cell foreign body reaction that was similar to the present findings around the suture material.

The use of bone suture anchors was reported as another method of abductor muscle reattachment to the greater trochanter in THA. Harwin reported the results of bone anchors used for abductor reattachment in cementless THA of 214 cases with the direct lateral approach [5]. There were several complications, such as anchor migration from the bone, progressive osteolysis of the greater trochanter, and pathological fracture of the greater trochanter. Because of these complications and the associated significantly increased cost, they did not recommend the use of bone anchors to repair the abductors in THA with the direct lateral approach.

Esenyel et al. reported that the Ethibond suture showed the lowest inflammatory reaction among three nonabsorbable suture materials at 6 weeks after operation in a rabbit model [6]. Ollivere et al. reported a case of foreign body granulomatous reaction associated with Fiberwire® which is braided blend of polyester and polyethylene suture used in Achilles tendon repair. The tissue reaction in the case was similar to the finding of polyethylene wear debris associated with osteolysis [8]. Therefore, the Ethibond suture has the lowest inflammatory tissue reaction and relatively high tension strength among nonabsorbable suture materials at the present time. However, number 5 Ethibond has the potential to cause osteolysis due to a foreign body granuloma, as in the present case. Therefore, Kundra et al. suggested that the use of a thinner polyester suture or a different material be recommended for abductor reattachment in THA to prevent the osteolysis of the greater trochanter [4]. Another nonabsorbable suture should not be challengingly used in reattachment of the hip abductors in THA as the long term result of its usage has not been reported. At the present, in terms of low inflammatory tissue reaction, the absorbable suture is recommended for the procedure although it has less mechanical properties than nonabsorbable ones. The osteolysis caused by number 5 Ethibond suture has to be kept in mind as a complication when it was used for repairing abductors through bone tunnels in THA.

## 4. Conclusion

A case of osteolysis of the greater trochanter caused by a foreign body granuloma associated with the number 5 Ethibond suture after THA with the direct lateral approach was presented. Although Ethibond suture has the lowest inflammatory tissue reaction among the nonabsorbable sutures, the number 5 Ethibond may cause osteolysis due to granuloma formation. The osteolysis caused by number 5 Ethibond suture has to be kept in mind as a complication when it was used for repairing abductors through bone tunnels in THA.
